# Comparative Morpho-Anatomical Studies of Two Philippine Endemic Species of *Amyema Tiegh*. (Loranthaceae)

**DOI:** 10.21315/tlsr2023.34.1.9

**Published:** 2023-03-31

**Authors:** Romeo M. Tubongbanua, Noe P. Mendez, Victor B. Amoroso

**Affiliations:** 1 Department of Biology, College of Arts and Sciences, College of Arts and Sciences, Central Mindanao University, University Town, Musuan, 8710 Bukidnon, Philippines; 2 Center for Biodiversity Research and Extension in Mindanao (CEBREM), Central Mindanao University, University Town, Musuan, 8710 Bukidnon, Philippines

**Keywords:** Clearing Technique, Free-Hand Technique, Hemiparasitic, Marilog Forest Reserve, Philippine Endemic

## Abstract

*Amyema* are epiphytic hemiparasitic plants on different types of woody host plants and are abundant in temperate, subtropical, and tropical regions. In Marilog Forest Reserve, Southern Philippines, two Philippine endemic species of *Amyema* were recorded, *viz*., *Amyema curranii* (Merr.) *Danser* and *A. seriata* (Merr.) Barlow. In this study, these two species were compared and examined for their morphology and anatomy. Data revealed that the two *Amyema* species are morphologically distinct, with *A. curranii* having lanceolate leaves, pink flowers, and red fruits, whereas *A. seriata* has obovate leaves, red flowers, and yellow fruits. For the morpho-anatomy, *A. curranii* has a single-layered epidermis, paracytic stomata, collateral open vascular bundles, the Eustele type of stele with pith at the center, and the inferior free central type with a hairy ovary wall. Meanwhile, *A. seriata* has a pinkish, single-layered epidermis, paracytic stomata, collateral open vascular bundles, a eustele type of stele with the presence of pith at the center, and an inferior free central ovary type. As a result, employing these species’ gross morphology and anatomy could scrutinise future evaluations and taxonomic placements.

**Highlights**
- The two *Amyema* species differ in the colour of flowers in which pink for *A. curranii* while red for *A. seriata*.
- The two species differ in haustorial attachment: *A. curranii* has several haustoria as it is creeping, and the ovary wall has the presence of hairs, while *A. seriata* has a solitary attachment and an ovary wall that is smooth.
- This is the first morpho-anatomical study of species in genus *Amyema* in the Philippines, which can be used to scrutinize taxa for species delineation.

## INTRODUCTION

*Loranthaceae*, also known as Showy Mistletoes, are perennial flowering plants that are known for their vivid inflorescence and enigmatic appearance (Devkota 2015). One of the genera in this family is *Amyema*, an epiphytic hemiparasitic plant (partially parasitic) on the xylem tissues of their host (Kuijt 1969; Calder & Bernhardt 1983; Kuijt 2009; Arruda *et al*. 2012), inhabiting a wide range of host plants (Devkota *et al*. 2011; Ogunmefun *et al*. 2015), and acquiring water, nutrients, and sugar while having the capability of photosynthesis (Norton & Carpenter 1998). *Amyema* is the second genus with high diversity in Loranthaceae, after *Psittacanthus*, which is the most specious (Kujit 2009).

The Philippine archipelago has a total of 24 *Amyema* species (Pelser *et al*. 2011), of which 19 are endemic to the country (Danser 1934; Barlow 1974; 1984; 1992). This genus is widely distributed from Southeast Asia to mainland Australia and the southwest Pacific (Danser 1934; Barlow 1974; 1984; 1992; Pelser & Barcelona 2013). Recently, three *Amyema* species have been described, *viz*., *A. nickrentii* Barcelona & Pelser in the Philippines (Pelser & Barcelona 2013), *A. lisae* Pelser and Barcelona also in the Philippines (Pelser *et al*. 2018), and *A. xiphophylla* Wege and Start in western Australia (Wege & Start 2020). Regardless of their detrimental effects on the host plant, they are still important in determining the floral diversity in forest ecosystems worldwide (Kujit 1964; Hawksworth 1983; Calder & Bernhardt 1983; Polhill & Wiens 1998; Devkota *et al*. 2011).

The Marilog Forest Reserve in Marilog District, Davao City, has a total land area of 63,000 ha. This area has a rich diversity of flora, which makes it one of the priority areas for biodiversity studies by Conservation International. Recently, several inventory studies on vascular flora in the area have been conducted (e.g., Acma et al. 2021; Coritico et al. 2022; Rufila et al. 2022). It is home to different parasitic plants, such as *Balanophora papuana* Schltr. (Balanophoraceae), *Amyema curranii (Merr.)* Danser and *A. seriata (Merr.)* Barlow (Loranthaceae), *Mitrastemon yamamotoi* Makino (Mitrastemonaceae), and *Christisonia scortechinii* Prain (Orobanchaceae), as documented by Acma *et al*. (2021). Of these, the two *Amyema* species were studied and examined for their gross morphology and anatomy.

## MATERIAL AND METHODS

### Botanical Fieldworks

**Figure 1:**
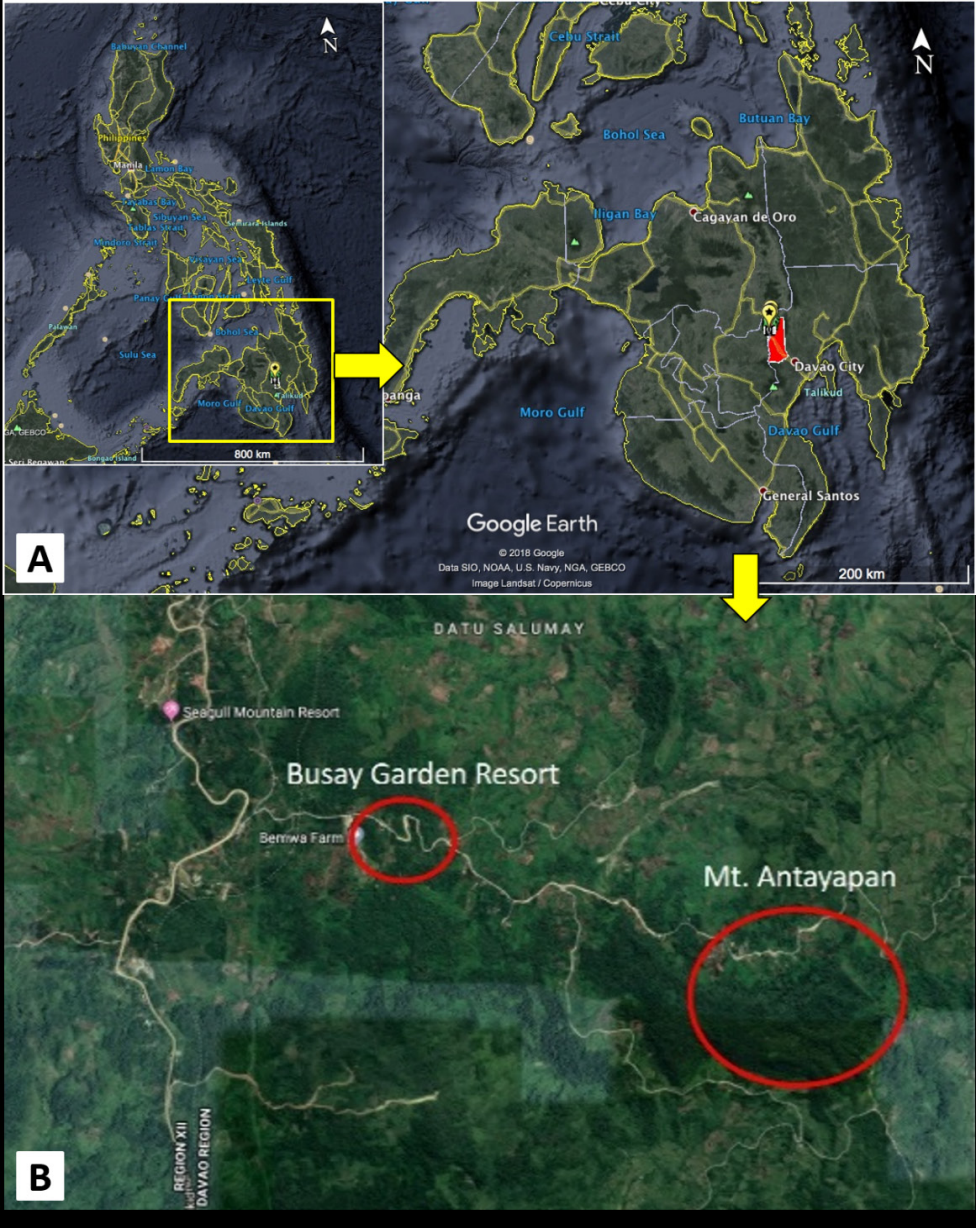
Study sites (A) Map of Mindanao Island (inset Philippine map); (B) Marilog District, Davao City, showing Mount Antayapan and Busay Garden Resort (in red circles). *Source*: (Google Earth)

Botanical field collections were carried out from August to November 2019.Necessary permits were obtained from the local people such as Gratuitous Permit (GP) from the Department of Environment and Natural Resources (DENR). The specimens of *A. curranii* were collected from Busay Garden Resort near the base of Mt. Malambo (a minor peak), while *A. seriata* was collected in Mt. Antayapan and Sitio Tribal Village, both in Brgy. Datu Salumay, Marilog District, Davao City, Philippines (Fig. 1). Repeated transect walks and opportunistic sampling were employed to survey and collect specimens. Moreover, characteristics of habitat, vegetation type, and documentation of host plants and anthropogenic disturbances in the areas were recorded.

### Identification of the Specimens

Specimens available at the Journal Storage (JSTOR) were used for the comparison, identification, and classification of *Amyema* species. For the purpose of verifying the morphological and anatomical characteristics of the plants, references from books, journals, and online databases, such as Pelser and Barcelona (2013) and The Global Plant List and Co’s Digital Flora of the Philippines by Pelser *et al*. (2011) forward, were utilised.

### Plant Measurements and Descriptions

The morphological characters of *A. curanii* and *A. seriata* were examined, documented, and described. Five plants were treated for each species. The lengths of the vegetative parts of living specimens were measured using a tape measure. Measurable features, such as plant height, stem length, length and width of leaf, length and diameter of the petiole, length and diameter of the haustorium, length of flowers and fruits, and detailed floral parts, were described and documented. The terminology of Wilson and Calvin (2006) was used in this study.

### Anatomical Analysis

Anatomy was done in the laboratory of the Department of Biology, Central Mindanao University, Musuan, Bukidnon. The procedure of Johansen (1940) for anatomical studies was followed. The free-hand technique was used to cut small pieces (about 1 mm long transverse sections) of the various plant parts. A clearing technique was done on the young leaves to study the venation pattern, stomatal type, and epidermal composition. Close-up views of the anatomy were taken using light and stereo microscopes.

## RESULTS AND DISCUSSION

### A. Gross Morphology

#### a. *Amyema curranii* (Fig. 2)

*Amyema curranii* stems creep around the host plant; leaves are lanceolate to oblanceolate, 10 cm–18 cm long. Inflorescence: pink, umbel-shaped, pedunculate inflorescence with a yellowish corolla tip. 10 to 15 flowers with at least 5 cm–5.5 cm long; fruits ovate, berry, aggregate, green when young, red when mature. It has a very close resemblance to *A. incarnatiflora* (Elmer) Danser, which has a pinkish, umbel-shaped, pedunculate inflorescence. They differ in the tip of the corolla, where *A. incarnatiflora* has a pinkish coloration from the base to the tip, while *A. curanii* has a yellowish corolla tip. Further, *A. curranii* has dimorphic leaves (Table 1).

#### b. *Amyema seriata* (Fig. 3)

*Amyema seriata* stems erect and mostly attached to the haustorium; leaves obovate to oblanceolate, 10–13 cm long. *Amyema seriata* stems erect and mostly attached to the haustorium; leaves obovate to oblanceolate, 10 cm–13 cm long. Inflorescence pedunculate, umbelled, and red, with dark red at the tip of the corolla, ca. 2.5 cm–3 cm long, with at least 6 to 8 flowers attached; fruits 2 cm long, berry-shaped, ovate, aggregated, green when young, and red when mature. *A. seriata* closely resembles *A. celebica* (Tiegh.) Danser, except for the latter having a red umbel, pedunculate inflorescence, and obovate leaves. They also differ at the tip of the corolla, where *A. celebica* has a yellowish tip and an outward fold when fully bloomed. It also has a pedicel attached to each flower (Table 1).

### B. Anatomy

**Figure 4:**
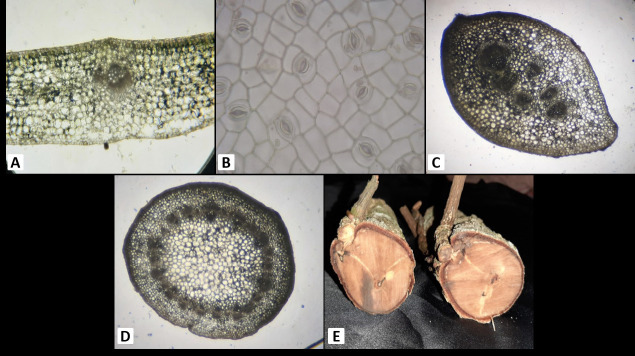
Anatomy of the vegetative parts *A. curranii*. (A) Cross section of leaf; (B) Cleared leaf epidermis; (C) Cross section of petiole; (D) Cross section of stem; (E) Transverse section of haustorium.

*Amyema curranii* leaf epidermis: single layer, pinkish; stomata are paracytic. Palisade mesophyll cells (5 layers–6 layers), spongy mesophyll cells (3 layers–5 layers), vascular bundle collaterals open, petiole epidermis single layer, cortex (12 layers–14 layers), and collateral open vascular bundles. Stem epidermis single layer, 10 layered–11 layered cortex, eustele with pith at the centre; several haustoria attached to the secondary xylem or the wood. Ovary inferior, free central, hairy (Fig. 4; Table 2).

**Figure 5:**
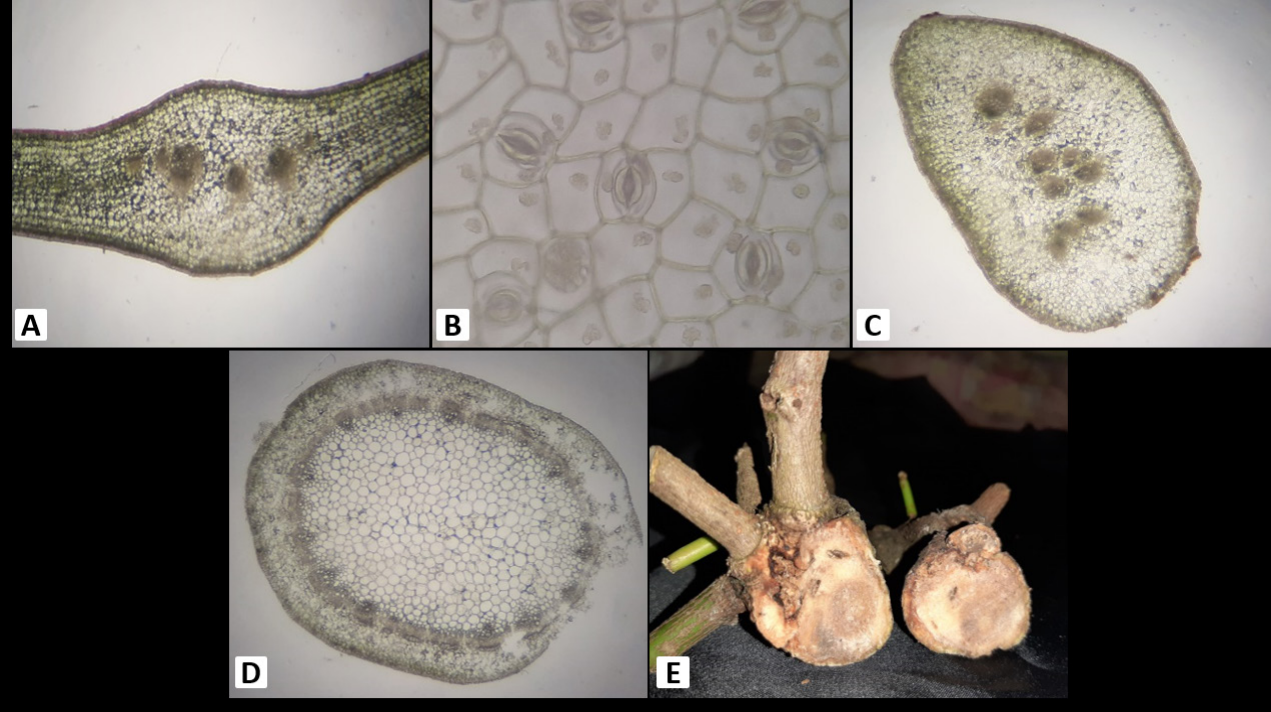
Anatomy of the vegetative parts *A. seriata*. (A) Cross section of leaf; (B) Cleared leaf epidermis; (C) Cross section of petiole; (D) Cross section of stem; (E) Transverse section of haustorium.

*A. seriata* has a single layer of leaf epidermis that is pinkish in colour and has paracytic stomata. Palisade mesophyll cells 4 layers–5 layers, spongy mesophyll cells 5 layers–6 layers, vascular bundle collateral open. Petiole epidermis, single layer, pinkish colour, cortex (10 layers–11 layers), vascular bundle collateral open. Stem epidermis has single layer, cortex 8 layers–10 layers; eustele stem has pith at the centre; haustorium is solitary and attached to the secondary xylem. The ovary is inferior, free central, and smooth (Fig. 5; Table 2).

According to Costa and Ceccantini (2015), the host plants should have a distinct thick-walled latewood as compared to that of the haustoria of parasitic plants. Haustorium evolution was complex in the aerial *Loranthaceae*, with multiple origins for each basic haustorial type (Wilson & Calvin 2006). They also tend to accumulate a large portion of the water reservoir in the woods, which can harm or kill the host plants (Hawksworth 1983). Some host plants also tend to resist the haustorium formation of Amyema by forming wound periderm or by means of changes in the host plant’s tissue (Yan, 1993). This can be shown in Figs. 4E and 5E, where both *Amyema* species have not fully penetrated the entire latewood or secondary xylem of the host plant.[Fig f6-tlsr-34-1-139]

## CONCLUSIONS

The flowers of the two *Amyema* species differ in colour, with *A. curranii* having pink flowers and *A. seriata* having red flowers. They also differ in haustorial attachment, where *A. curranii* has several haustoria as it is creeping, *while A. seriata* has a solitary attachment, and ovary wall, where *A. curranii* has the presence of hairs, whereas *A. seriata* is smooth.

## FUTURE SCOPE

Conservation and protection of the Marilog Forest Reserve should be implemented due to the increasing forest and habitat disturbances caused by several anthropogenic activities that place these plants at risk of vulnerability or depletion. Anatomical studies using other techniques are also suggested to carefully record the differences in their parts.

## Figures and Tables

**Figure 2:**
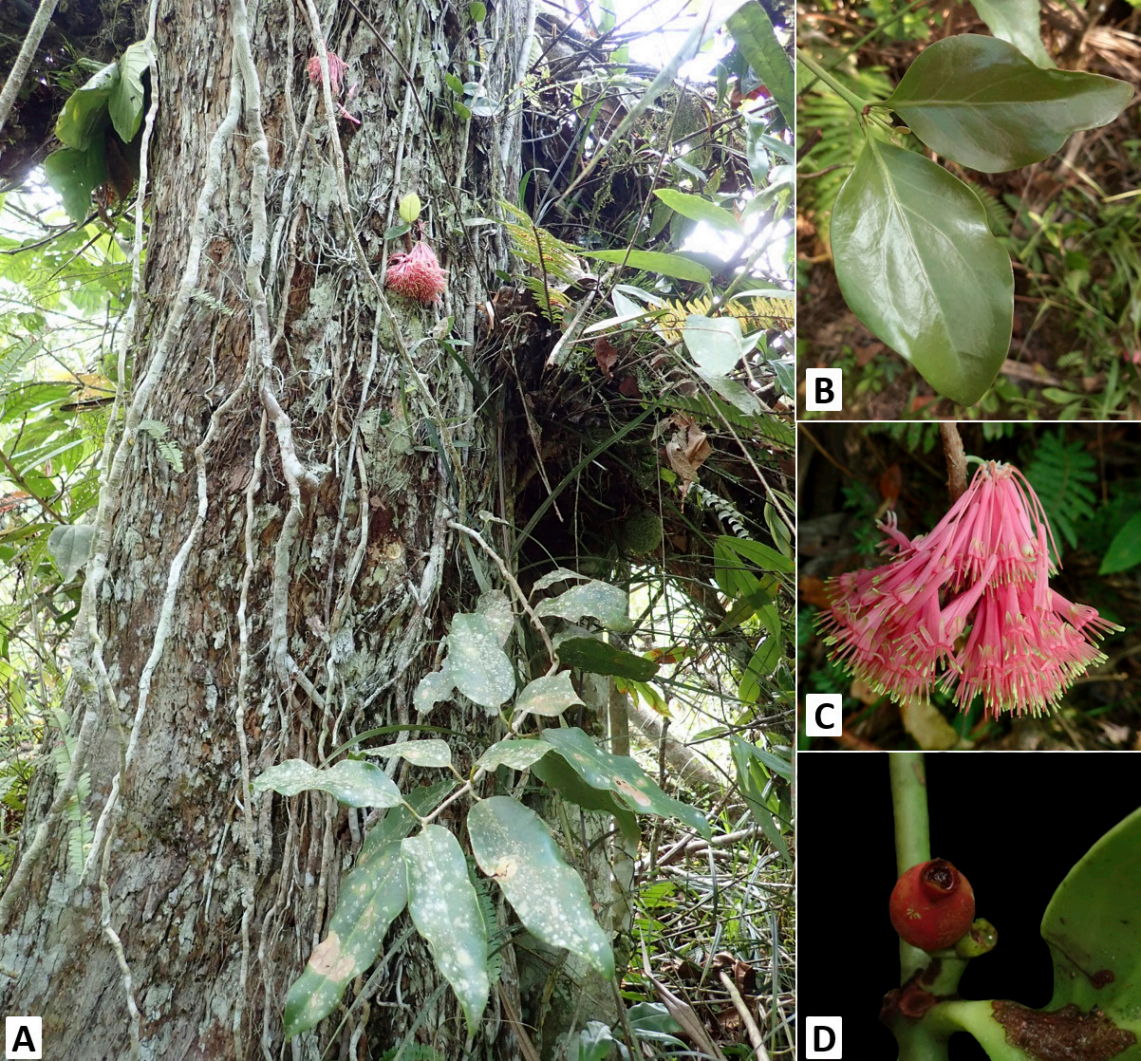
Morphology of *Amyema curranii* (Merr.) Danser (A) Habit; (B) Leaves; (C) Inflorescence; (D) Fruit.

**Figure 3:**
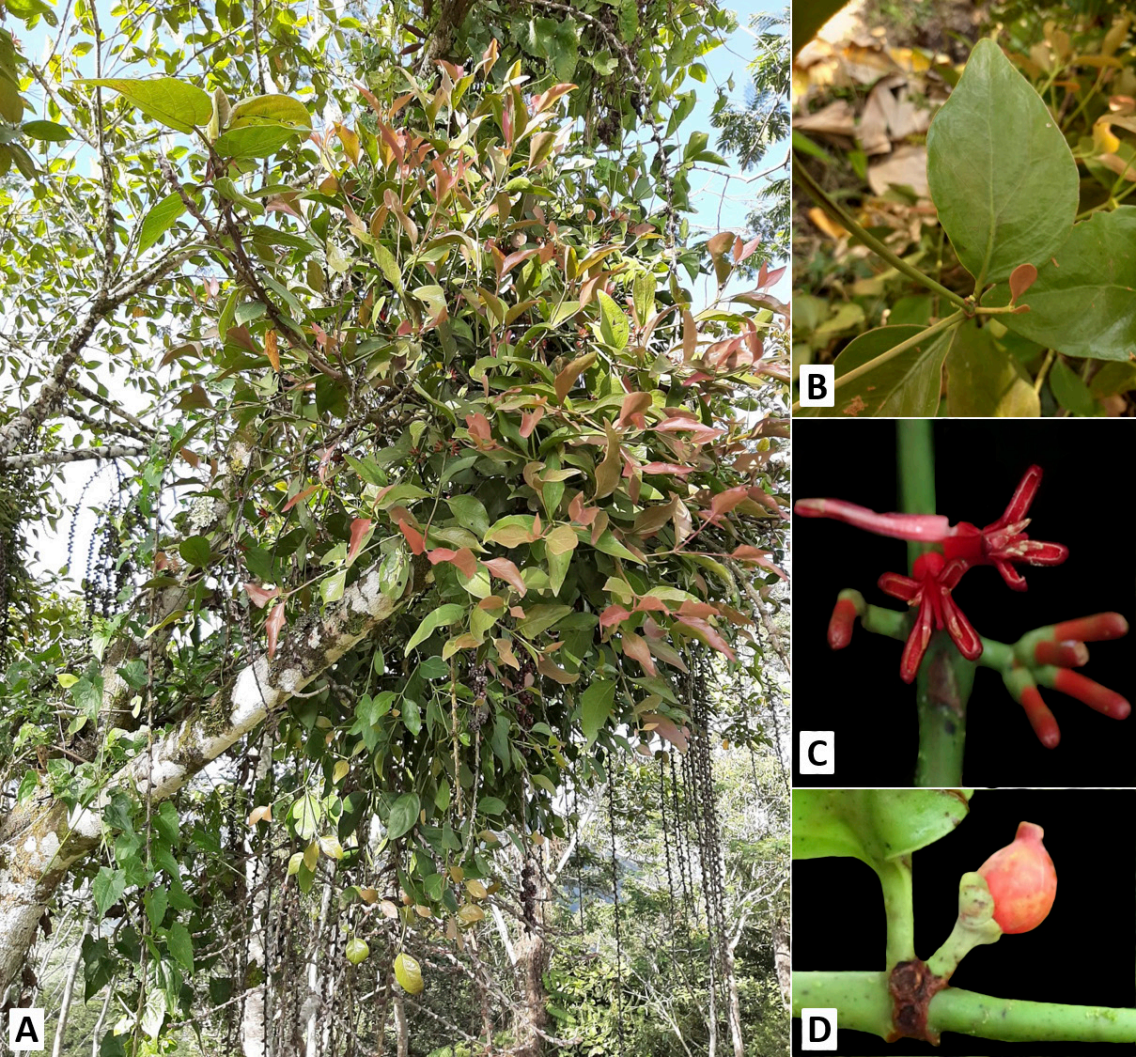
Morphology of *Amyema seriata* (Merr.) Barlow. (A) Habit, (B) Leaves, (C) Inflorescence, (D) Fruit.

**Figure 6 f6-tlsr-34-1-139:**
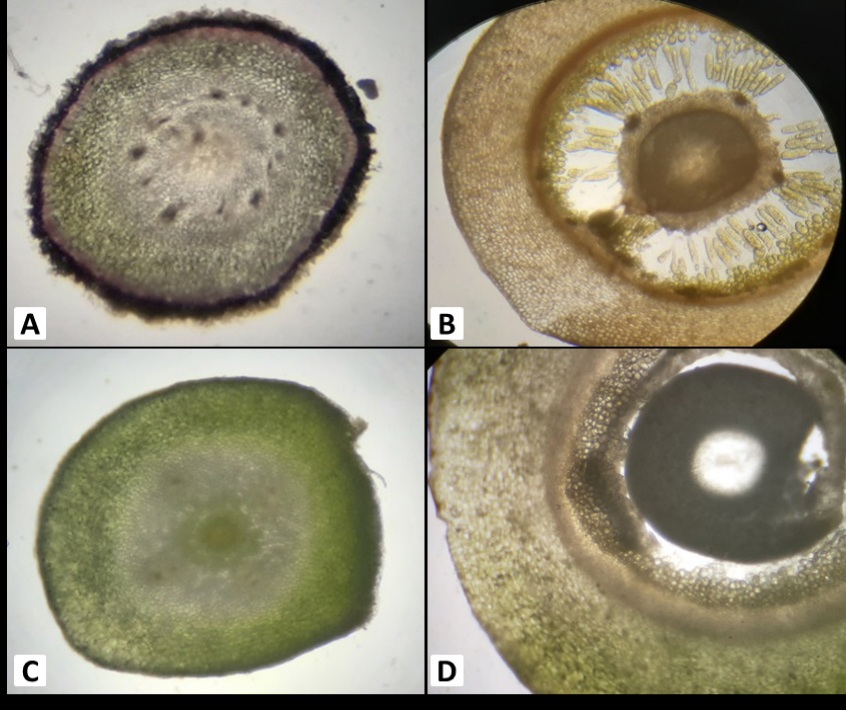
Anatomy of the ovary (A) Transverse section of the young ovary of *A. curranii*, (B) Transverse section of mature ovary of *A. curranii*, (C) Transverse section of the young ovary of *A. seriata*, (D) Transverse section of mature ovary of *A. seriata*.

**Table 1:** Comparative morphological characters of the two *Amyema* species.

Gross Morphology	*A. curranii*	*A. seriata*
**Leaves**		
Arrangement	Opposite	Opposite
Shape	Oblanceolate/lanceolate	Oblanceolate to obovate; 4–5 in long
Colour	Brownish green (Young), Green (Mature)	Brownish green (Young), Green (Mature)
Texture	Smooth	Smooth
Venation pattern	Netted	Netted
Base	Obtuse	Obtuse
Apex	Acute	Acute
Margin	Simple	Simple
Length	10 cm–18 cm	10 cm–12 cm
Width	5 cm–10 cm	5 cm–8 cm
**Stem**	Creeping around the host plant	Erect mostly attached to haustorium
**Flower**		
Inflorescence	Umbel	Umbel
Corolla (petal)	Pink with yellowish colour on the apex	Red with deep reddish colouration at the apex
Length	5 cm–5.3 cm	2 cm–2.5 cm
Peduncle	Present	Present
Pedicel	Present	Present
**Fruit**		
Type	Berry	Berry
Placentation	Aggregate	Aggregate
Texture	Smooth	Smooth
Colour	Green when young, Red when ripe	Green when young, Red when ripe
Peduncle	Present	Present
Pedicel	Present	Present
Shape	Obovate	Obovate
Length	1 cm–1.5 cm	0.8 cm–1.2 cm

**Table 2:** Anatomy of vegetative and reproductive parts of the two *Amyema* species.

Anatomy	*A. curranii*	*A. seriata*
**Leaf**		
Epidermis	Single layer, pinkish colour	Single layer, pinkish colour
Stomata	Paracytic	Paracytic
Palisade mesophyll	5 layers–6 layers	4 layers–5 layers
Spongy mesophyll	3 layers–5 layers	5 layers–6 layers
Vascular bundles	Collateral open	Collateral open
**Petiole**		
Epidermis	Single layer	Single layer
Cortex	12 layers–14 layers	10 layers–11 layers
Vascular bundle	Collateral open	Collateral open
**Stem**		
Epidermis	Single layer	Single layer
Cortex	10 layers–11 layers	8 layers–11 layers
Stele	Eustele	Eustele
Pith	Present	Present
**Haustorium**		
Type	Several, attached to the secondary xylem or wood	Solitary, attached to the secondary xylem or wood
**Ovary**		
Type	Hairy, inferior, free central	Smooth, inferior, free central
